# Effects of Probiotic Supplementation on Performance Retention During Detraining and Recovery After Simulated Return-to-Training Stress in College Volleyball Players: An Exploratory Randomized, Single-Blind, Placebo-Controlled Trial

**DOI:** 10.3390/nu18193203

**Published:** 2026-09-28

**Authors:** Jin Liu, Kai Zhao, Chenzhe Liu, Zhichen Liu, Wei Jiang

**Affiliations:** 1 Department of Exercise Physiology, School of Sport Science, Beijing Sport University, Beijing 100084, China; liujin10222001@163.com (J.L.); 2025210322@bsu.edu.cn (Z.L.); 2 Laboratory of Sports Stress and Adaptation of General Administration of Sport, Beijing 100084, China; 2326@bsu.edu.cn; 3 China Volleyball Sport College, Beijing Sport University, Beijing 100084, China; 2430@bsu.edu.cn

**Keywords:** probiotics, volleyball players, detraining, lower-limb power, post-exercise recovery, gut microbiota, gut–muscle axis

## Abstract

**Background:** The effects of probiotic supplementation on performance and recovery during training transitions remain uncertain. This exploratory trial examined the effects of probiotic supplementation on performance, recovery, and gut microbiota in national-level university volleyball players. **Methods:** Twenty-eight players were randomized to receive a multi-strain probiotic or placebo (n = 14 per group) for 8 weeks, comprising regular training, tapering and competition, and reduced training. Performance and gut microbiota were assessed before and after supplementation. Participants then completed 200 weighted vertical jumps as a simulated return-to-training stressor, with recovery monitored for 48 h. **Results:** Before accounting for multiple comparisons, the probiotic group showed smaller declines in shuttle-test performance and standing and approach vertical jump height across the intervention period, with no significant between-group differences in maximal-strength or Wingate outcomes. Following the simulated return-to-training stressor, the probiotic group showed lower muscle soreness and creatine kinase responses and smaller reductions in selected strength-recovery and rate-of-force-development measures. After accounting for multiple comparisons, only lower muscle soreness and a smaller reduction in non-dominant knee extension maximum work at 180°/s at 24 h remained significant. Chao1 richness decreased more in the probiotic group, whereas changes in Simpson diversity and the relative abundances of Firmicutes, Lachnospiraceae, and Bifidobacteriaceae did not differ between groups. **Conclusions:** Eight weeks of multi-strain probiotic supplementation was associated with selected differences in performance and recovery during a training transition. However, after accounting for multiple comparisons, the evidence was limited to lower muscle soreness and a smaller reduction in non-dominant knee extension maximum work at 24 h. These exploratory findings require confirmation in larger, prospectively registered trials with prespecified outcomes.

## 1. Introduction

Volleyball is a high-intensity intermittent sport that demands frequent execution of explosive actions such as spiking, blocking and defensive transitions, placing exceptional demands on lower-body power and rapid recovery capacity [1]. Elite-level volleyball players perform approximately 200 jumps per match on average [2], underscoring that superior lower-limb strength is fundamental to competitive performance. As the competitive level of volleyball has risen, match schedules have become increasingly condensed, and elite athletes in professional leagues or collegiate sports often face multiple matches within a 48-h window. This frequent eccentric loading can induce delayed-onset muscle soreness (DOMS) and lower-limb muscle fatigue [3]; inadequate recovery between bouts not only impairs subsequent power output but also increases the risk of injury [4].

Training and competition in volleyball typically follow a seasonal periodization model, comprising pre-season preparation, in-season maintenance and a post-season transition phase with reduced load. The pre-season phase focuses on developing explosive power, the in-season phase emphasizes rapid recovery and the low-load post-season period often leads to detraining. Athletes frequently experience training interruptions due to the off-season, injury or holidays [4]. Although such breaks can be beneficial for systemic recovery, the high-intensity workload during the initial phase of return to training can easily lead to fatigue and exercise-induced muscle damage (EIMD). Consequently, volleyball athletes commonly face three major challenges: insufficient recovery time between matches, detraining and associated adaptation loss following the competitive season, and an elevated risk of fatigue and injury during the initial return-to-training period [5]. Evidence indicates that periods of training cessation or substantial training reduction can diminish muscle contractile capacity, can reduce neuromuscular activation and may promote atrophy of fast-twitch muscle fibers, collectively resulting in decreased rate of force development (RFD) and explosive power [6,7]. These detrimental adaptations can emerge within as little as two weeks, particularly in well-trained strength athletes [8], impairing athletic performance and increasing injury risk upon resumption of training. Existing strategies, such as modified training programs [9] or nutritional interventions [10], have demonstrated mixed or context-dependent effects, highlighting the need for targeted nutritional interventions to mitigate strength loss during training breaks and to accelerate functional recovery upon return to training.

The emerging gut–muscle axis theory proposes that gut microbiota and their metabolites, such as short-chain fatty acids (SCFAs) and secondary bile acids, regulate skeletal muscle mass, strength and function through multiple mechanisms. These include modulating systemic inflammatory responses, ameliorating oxidative stress, enhancing mitochondrial function, regulating energy metabolism pathways and influencing intracellular signaling in muscle cells [11,12]. In recent years, probiotics—defined as live microorganisms that confer a health benefit on the host when administered in adequate amounts—have emerged as potential ergogenic aids. Research suggests that probiotics may help athletes maintain strength and accelerate recovery by optimizing energy metabolism and attenuating inflammatory responses [13]. Several studies have demonstrated that specific strains can enhance muscle strength and power [14], mitigate exercise-induced muscle damage and shorten recovery time [15,16,17]. For example, Lactobacillus paracasei PS23 attenuated post-exercise declines in muscle strength and reduced inflammatory markers [17]. Combined probiotic and vitamin D supplementation also improved power output in martial arts athletes [18], and synbiotic intervention improved upper-body explosive strength in elite swimmers [19]. However, research has primarily focused on triathlon [15] or resistance training [20], and investigations into the effects of probiotic supplementation on maintaining lower-limb strength and facilitating recovery following exercise-induced muscle damage in volleyball athletes remain scarce. Unlike prior studies that mainly examined stable training periods or non-volleyball populations, the present study focused on a practically relevant yet underexplored transition phase in athletes: the period spanning post-season detraining and simulated return-to-training stress. By evaluating both detraining-related performance loss and recovery following a standardized EIMD protocol, this exploratory trial aimed to examine the effects of probiotic supplementation on volleyball-relevant performance and recovery.

## 2. Materials and Methods

### 2.1. Participants

This study included 28 national-level university volleyball players (National Level 1 or above) from Beijing Sport University, all with ≥6 years of systematic training. The study protocol was approved by the Ethics Committee of Beijing Sport University (protocol code 2024220H; approved on 1 November 2024) and was conducted in accordance with the principles of the Declaration of Helsinki. Participants were prospectively recruited and enrolled in the study. Inclusion criteria were: no use of prebiotics, probiotics, synbiotics or fermented foods (e.g., yogurt) before the study, and ≥6 h/week of training. Exclusion criteria were: gastrointestinal diseases, musculoskeletal injuries in the past 6 months, probiotic use in the past 3 months, antibiotic use in the past month, or hypersensitivity to probiotics. All participants provided written informed consent. Using computerized block randomization, participants were assigned to probiotic (P) or placebo (C) groups (n = 14 each). Each group comprised 4 males and 10 females: 7 National Elite and 7 First-Grade athletes. Applying the participant classification framework of McKay et al. [21], the cohort corresponds to Tier 3 (Highly Trained/National Level). Baseline anthropometrics showed no significant differences in age (P: 20 ± 1 years, C: 20 ± 2 years), height (P: 184 ± 8 cm, C: 183 ± 8 cm) or body mass (P: 73 ± 11 kg, C: 73 ± 11 kg, all *p* > 0.05). The trial was subsequently registered in the Chinese Clinical Trial Registry (ChiCTR; registration number: ChiCTR2600127154; registration date: 25 June 2026), after participant enrollment had been completed. Thus, the trial was registered retrospectively, whereas participant recruitment and enrollment were prospective. The original ethics documentation did not formally specify primary and secondary outcomes or a detailed statistical analysis plan.

### 2.2. Study Design

This 8-week randomized, single-blind, placebo-controlled trial spanned the late competitive season and the post-competition detraining phase of the athletes’ annual periodization cycle. Specifically, the 8-week supplementation period consisted of three successive phases: an initial 4-week regular training phase, followed by a 2-week combined taper and competition phase, and finally a 2-week detraining phase, during which participants’ usual structured volleyball training was substantially reduced rather than completely discontinued. Supplementation continued throughout all three phases. Weekly training load was calculated using session rating of perceived exertion (RPE) and duration, and the International Physical Activity Questionnaire (IPAQ) was used to assess daily activity during the detraining period (Supplementary Material, Tables S1 and S2). No between-group differences in training load or off-training activity were observed. Pre-supplementation (baseline, at the start of week 1) and post-supplementation (at the end of week 8, i.e., after the 2-week detraining phase) tests used identical protocols. Immediately after the post-supplementation test, athletes completed a standardized muscle-damaging exercise protocol (200 weighted vertical jumps) to simulate return-to-training stress. Acute post-exercise responses were assessed immediately after the protocol, and recovery from exercise-induced muscle damage was monitored at 24 and 48 h. The complete study timeline and measurement schedule are presented in Figure 1.

### 2.3. Outcomes

The outcomes were categorized as co-primary and secondary outcomes for the purposes of the present analysis; this categorization was not prospectively prespecified in the original ethics documentation. The co-primary outcomes were: (1) lower-limb strength and explosive performance following the reduced-training period, assessed by IMTP and isokinetic knee strength; (2) post-exercise recovery and muscle-damage outcomes following the standardized muscle-damaging exercise protocol, assessed by muscle soreness, serum CK, and recovery of strength and RFD at 24 and 48 h, including SJ height, relative peak force, and RFD. Secondary outcomes included sport-specific agility, vertical jump performance, Wingate test parameters, and gut microbiota composition.

### 2.4. Supplementation Protocol

Probiotics and placebo (2 g per sachet, powder) were supplied by Beijing Scitop Bio-tech Co., Ltd. (Beijing, China). The P group received a daily multi-strain probiotic containing Lactobacillus casei Zhang (≥8 × 109 CFU), Bifidobacterium lactis V9 (≥6 × 109 CFU) and Lactobacillus plantarum P-8 (≥6 × 109 CFU). The probiotic and placebo had the same batch number (24090101), although their production dates differed, and were matched in appearance, taste, and packaging. Participants were blinded to treatment allocation throughout the intervention. Personnel responsible for supplement allocation and distribution were aware of group assignments, whereas outcome assessors and laboratory personnel were blinded. Therefore, the study was classified as single-blind. One sachet was taken after breakfast for 8 weeks. Participants were instructed to maintain their habitual dietary patterns throughout the intervention and to follow the predefined dietary avoidance list, which included probiotics, prebiotics, synbiotics, and fermented foods (including yogurt). No specific dietary intervention or prescribed diet was provided. A ≥2-h interval was required if antibiotics were used. Adherence was monitored daily through direct communication with the research team and cross-checked against the number of empty intervention sachets returned weekly. Adherence was 100% (14/14) in both the probiotic and placebo groups, and all 28 participants completed the intervention without withdrawal due to non-adherence.

### 2.5. Body Composition

Body height was measured using a standardized stadiometer. Body mass, skeletal muscle mass, body fat mass, soft lean mass, fat-free mass, body fat percentage, and waist-to-hip ratio were obtained in the morning under fasting conditions using a bioelectrical impedance analyzer (InBody 720, InBody Co., Ltd., Seoul, Republic of Korea). Anthropometric and body-composition measurements were performed at baseline and again at the end of the 8-week intervention under the same standardized conditions.

### 2.6. Vertical Jump Test

Vertical jump height was measured for standing (SVJ) and approach (AVJ) jumps using a vertical jump apparatus (MGQ-003, Beijing Zhongti Tongfang Sports Technology Co., Ltd., Beijing, China). After a standardized warm-up, participants completed three trials per test. The SVJ required a maximal jump from a static, shoulder-width stance. The AVJ allowed a 2–3 step approach. Jump height was calculated as the difference between the highest vane touched during the jump and the participant’s standing reach height. The greatest jump height across the three trials was used for analysis.

### 2.7. Standing Long Jump Test

The standing long jump (SLJ) was conducted on an outdoor athletic field in a marked sandpit. Participants stood behind the take-off line and performed a two-legged jump for maximal horizontal distance. Each participant performed two trials, and the best result was recorded.

### 2.8. Half-Meter Shuttle Test

The half-meter shuttle test was performed on one half of a standard volleyball court. Participants started at the baseline midpoint, sprinted to the far right baseline and back, right-front attack zone and back, net center and back, left-front attack zone and back, and far left baseline and back. Each marker plate was touched. Total time was recorded; two trials were performed and the best time was used for analysis. A schematic representation of the test is provided in Supplementary Figure S1.

### 2.9. Wingate Anaerobic Test

A 30-s Wingate test (Monark 894E, Monark Exercise AB, Vansbro, Sweden) was conducted at a resistance of 7.5% of body mass. After a 5-min warm-up (60 rpm with two maximal sprints), participants performed a 30-s all-out sprint. Peak power, mean power and fatigue index were recorded. Fatigue index (%) = [(peak power − minimum power)/peak power] × 100%.

### 2.10. Isometric Mid-Thigh Pull (IMTP) Test

Maximal isometric strength was assessed using a force platform (KWFP6040-10K, Changzhou Kunwei Sensing Technology Co., Ltd., Changzhou, China). The barbell was fixed at mid-thigh height with the knee and hip slightly flexed (knee angle ≈ 125°). Participants performed three maximal pulls, each sustained for 3–5 s. The highest absolute peak force (N) across the three trials was recorded. IMTP peak force ratio was calculated as peak force divided by body weight force: (absolute peak force − body weight force)/body weight force, where body weight force was calculated as body mass multiplied by gravitational acceleration (9.81 m·s^−2^). The highest IMTP peak force ratio across the three trials was used for statistical analysis. The IMTP peak force ratio is dimensionless.

### 2.11. Squat Jump (SJ) Test

Squat jumps were performed on a force platform (KWFP6040-10K, Changzhou Kunwei Sensing Technology Co., Ltd., Changzhou, China) from a static 90° knee position without counter-movement. Jump height, relative peak force, and peak RFD were recorded. Three trials with 1-min rest were performed, and the best performance was used.

### 2.12. Isokinetic Knee Strength Test

Knee extension strength was measured using an isokinetic dynamometer (ISOMED 2000, D&R Ferstl GmbH, Hemau, Germany) at angular velocities of 60°/s and 180°/s. For the purposes of this study, the dominant limb was defined as the limb with the greater peak torque at 60°/s during the baseline isokinetic knee strength assessment, and the contralateral limb was classified as the non-dominant limb. Participants performed three maximal repetitions at each velocity. Relative peak torque and relative maximum work were recorded for both the dominant and non-dominant limbs. The emphasis on the non-dominant limb reflects the pattern observed in the present data rather than a prespecified focus on this limb.

### 2.13. Muscle-Damaging Exercise Protocol

Following the reduced-training period, participants completed a standardized simulated return-to-training stressor consisting of 200 weighted jumps (10% of body mass, 10 × 20 maximal vertical jumps, 4 s between jumps, 2 min rest between sets) [22]. The protocol was designed to induce acute fatigue and exercise-induced muscle damage through a controlled exercise stressor intended to approximate the physical demands that may be encountered during the initial return to training. It was not intended to replicate or replace a real-world retraining program. During the 48-h recovery monitoring period, participants were instructed to avoid additional exercise, massage, cryotherapy or anti-inflammatory medications.

### 2.14. Post-Exercise Recovery and Muscle-Damage Assessment

#### 2.14.1. Performance Tests

The IMTP, SJ and isokinetic knee strength tests were performed before the muscle-damaging exercise protocol (pre-exercise) and at 24 and 48 h post-exercise, following the same methodologies described above. A complete overview of all measurements and their time points is provided in Figure 1.

#### 2.14.2. Rating of Perceived Exertion and Muscle Soreness

The Borg RPE scale (6–20) was administered once, immediately after completion of the 200 weighted vertical jumps, to assess perceived exercise intensity. Muscle soreness was assessed using a 10-cm visual analog scale (VAS; 0 = no pain, 10 = severe pain) at pre-exercise and at 0, 24 and 48 h post-exercise. Participants marked their soreness level, and the distance (cm) from the origin was recorded.

#### 2.14.3. Blood Analysis

Fasting venous blood (5 mL) was collected from the antecubital vein before the muscle-damaging exercise protocol, 24 h and 48 h post-exercise. Samples were centrifuged and serum stored at −80 °C. Serum creatine kinase (CK) was measured using a commercial ELISA kit (Shanghai Jianglai Biotechnology Co., Ltd., Shanghai, China).

### 2.15. Gut Microbiota Analysis

Participants avoided microbiota-influencing foods (e.g., probiotics, fermented foods, high-sugar diets) for 24 h prior to fecal collection. Morning samples (3–5 g) were self-collected aseptically, immediately flash-frozen in liquid nitrogen, transported to the laboratory within 1 h and stored at −80 °C. DNA was extracted, and the 16S rRNA V3–V4 region was amplified and sequenced on an Illumina MiSeq platform (Illumina, San Diego, CA, USA). Sequencing data were analyzed using QIIME 2. Alpha diversity and taxonomic composition (phylum and family levels) were analyzed.

### 2.16. Statistical Analysis

All analyses were performed using SPSS 27.0 (IBM Corp., Armonk, NY, USA), with data expressed as mean ± SEM, except for age, height, and body mass, which are presented as mean ± SD. For outcomes expressed as raw values (WAnT peak power, mean power, fatigue index, IMTP peak force ratio, isokinetic strength, VAS) and body-composition outcomes assessed before and after the intervention, repeated-measures ANOVA was used to evaluate group × time effects, followed by Bonferroni-adjusted pairwise comparisons where appropriate. For detraining outcomes expressed as percentage changes from baseline (such as shuttle test, SLJ, SVJ, and AVJ), between-group comparisons were performed using independent-samples *t*-tests. For post-exercise recovery and muscle-damage outcomes assessed at 24 and 48 h, expressed as percentage changes from pre-exercise (CK, IMTP, SJ, and isokinetic strength) and for baseline anthropometric characteristics, between-group comparisons were performed using independent-samples *t*-tests or Mann–Whitney U tests according to data distribution and variance homogeneity. The Borg RPE score recorded immediately after the muscle-damaging protocol was compared between groups using an independent-samples *t*-test. For microbiome outcomes, group × time interactions were evaluated using repeated-measures ANOVA. Exploratory within-group pre–post comparisons were performed using paired *t*-tests. A two-tailed *p* < 0.05 was considered statistically significant. Effect sizes were estimated using Cohen’s d (small: 0.2, medium: 0.5, large: 0.8), and 95% confidence intervals (95% CIs) were calculated for all between-group mean differences. For repeated-measures ANOVA, partial eta squared (ηp^2^) was used to characterize the magnitude of group × time interactions where applicable. Bonferroni adjustment was applied to post hoc pairwise comparisons following significant omnibus effects. Given the exploratory nature of this trial and the number of outcomes assessed, post hoc sensitivity analyses were performed using the Holm procedure. Holm adjustment was applied separately to the between-group performance and recovery comparisons presented in Figure 2, Figure 3 and Figure 4, the two group × time interaction tests for alpha diversity, and the three group × time interaction tests for taxonomic composition. These sensitivity analyses were not prospectively prespecified.

## 3. Results

### 3.1. Participant Characteristics and Body Composition

Participant anthropometric characteristics before the 8-week intervention are presented in Table 1. No significant between-group differences were observed in any anthropometric or body-composition variable. Body composition before and after the intervention is summarized in Supplementary Table S5. No significant group × time interactions were observed for body mass or any other body-composition variable (all *p* > 0.05), indicating no statistically significant between-group differences in pre-to-post changes.

### 3.2. Effects of Probiotic Supplementation on Sport-Specific Performance

The impact of the 8-week probiotic intervention on sport-specific performance is summarized in Figure 2. Compared with pre-intervention values, the sport-specific performance declined from pre- to post-intervention, as reflected by increased half-meter shuttle test time (Figure 2A) and decreased SLJ distance (Figure 2B), SVJ height (Figure 2C) and AVJ height (Figure 2D). Absolute pre- and post-intervention values for the sport-specific performance outcomes are presented in Supplementary Table S3.

Following the intervention, the increase in half-meter shuttle test completion time was significantly smaller in the P group than in the C group (P: +2.34 ± 0.43%, C: +4.04 ± 0.69%, Cohen’s d = −0.79, 95% CI: −3.29 to −0.11, *p* < 0.05, Figure 2A). Compared with the C group, the P group showed a smaller percentage decrease in SVJ height (P: −5.95 ± 0.89%, C: −9.42 ± 1.39%, Cohen’s d = 0.80, 95% CI: 0.08 to 6.85, *p* < 0.05, Figure 2C) and AVJ height (P: −9.10 ± 1.22%, C: −13.02 ± 1.46%, Cohen’s d = 0.78, 95% CI: 0.19 to 7.65, *p* < 0.05, Figure 2D). No significant between-group difference was observed for the SLJ (*p* > 0.05).

### 3.3. Effects on Lower-Limb Strength and Anaerobic Capacity

#### 3.3.1. Isometric Mid-Thigh Pull (IMTP)

The effect of supplementation on IMTP peak force ratio is shown in Figure 2H. Repeated-measures ANOVA revealed a significant main effect of time for IMTP peak force ratio (F(1, 26) = 33.99, *p* < 0.001, ηp^2^ = 0.56), indicating significant reductions in both groups following detraining. However, the group × time interaction was not significant, indicating that probiotic supplementation did not differentially affect maximal isometric strength during detraining.

#### 3.3.2. Wingate Test

Wingate test results before and after the 8-week intervention are shown in Figure 2E–G. Repeated-measures ANOVA revealed a significant main effect of time for relative peak power (F(1,26) = 40.75, *p* < 0.001, ηp^2^ = 0.61), relative mean power (F(1,26) = 84.20, *p* < 0.001, ηp^2^ = 0.76), and fatigue index (F(1,26) = 63.52, *p* < 0.001, ηp^2^ = 0.71), indicating changes over time in the Wingate outcomes. However, the group × time interaction was not significant for any WAnT parameter (all *p* > 0.05), indicating that probiotic supplementation did not differentially affect Wingate performance during detraining.

#### 3.3.3. Isokinetic Knee Strength

Isokinetic knee strength was assessed pre- and post-intervention at 60°/s and 180°/s, with relative peak torque and relative maximum work recorded for both limbs (Table 2). Repeated-measures ANOVA revealed significant main effects of time for all isokinetic measures (all *p* < 0.001), indicating post-test declines across all conditions. However, the group × time interactions were not significant for any measure (all *p* > 0.05), indicating that probiotic supplementation did not differentially affect isokinetic knee strength during detraining.

### 3.4. Post-Exercise Recovery and Muscle-Damage Outcomes

#### 3.4.1. Perceived Exertion, Muscle Soreness, and Creatine Kinase

Immediately after completion of the muscle-damaging exercise protocol, the mean RPE was 17.93 ± 0.26 in the P group and 18.07 ± 0.23 in the C group. Every participant rated the effort at ≥17 on the Borg 6–20 scale, indicating high perceived exertion. No statistically significant between-group difference in perceived exertion was detected (Cohen’s d = −0.15; 95% CI: −0.85 to 0.55; *p* > 0.05).

Perceived muscle soreness (DOMS) was monitored at pre-exercise, immediately post-exercise, and at 24 h and 48 h after the muscle-damaging exercise protocol, whereas serum CK was assessed pre-exercise and at 24 and 48 h post-exercise (Figure 3). Repeated-measures ANOVA revealed a significant main effect of time for VAS (F(3,66) = 94.59, *p* < 0.001, ηp^2^ = 0.811), reflecting increased muscle soreness following the exercise protocol in both groups. The group × time interaction did not reach statistical significance. However, between-group comparisons at individual time points revealed that the P group reported significantly lower muscle soreness than the C group at 24 h post-exercise (P: 4.26 ± 0.21 cm, C: 5.66 ± 0.40 cm, Cohen’s d = −1.17, 95% CI: −2.29 to −0.51, *p* < 0.05, Figure 3A). No significant between-group differences were observed at the remaining time points (*p* > 0.05).

Additionally, the percentage change in CK levels after the muscle-damaging exercise protocol was analyzed (Figure 3B). The increase in CK was significantly attenuated in the P group compared with the C group at both 24 h (P: 33.46 ± 5.11%, C: 50.33 ± 6.16%, Cohen’s d = −0.89, 95% CI: −32.56 to −1.18, p < 0.05) and 48 h post-exercise (P: 19.82 ± 4.06%, C: 31.46 ± 3.76%, Cohen’s d = −0.87, 95% CI: −22.49 to −0.79, p < 0.05, Figure 3B). Absolute values for the post-exercise recovery and muscle-damage outcomes at each measurement time point are presented in Supplementary Table S4.

#### 3.4.2. IMTP Recovery

IMTP performance was assessed pre-exercise and at 24 h and 48 h post-exercise (Figure 3C). The percentage reduction in the IMTP peak force ratio was significantly smaller in the P group compared with the C group at 24 h post-exercise (P: −6.07 ± 0.71%, C: −9.53 ± 1.26%, Cohen’s d = 0.90, 95% CI: 0.63 to 6.29, *p* < 0.05, Figure 3C). Although the P group still showed a smaller reduction at 48 h, the difference was not statistically significant (*p* > 0.05).

#### 3.4.3. Squat Jump (SJ) Recovery

SJ tests were performed pre-exercise and at 24 h and 48 h post-exercise (Figure 3D–F). The reduction in SJ relative peak force was smaller in the P group than in the C group at 24 h (P: −4.42 ± 0.50%, C: −6.92 ± 1.07%, Cohen’s d = 0.86, 95% CI: 0.05 to 4.95, *p* < 0.05) and 48 h (P: −2.62 ± 0.44%, C: −4.62 ± 0.71%; 95% CI: 0.26 to 3.73; Cohen’s d = 0.97, *p* < 0.05, Figure 3D). The decrease in RFD was significantly smaller in the P group at 24 h (P: −12.66 ± 1.28%, C: −18.34 ± 2.13%, Cohen’s d = 0.86, 95% CI: 0.81 to 10.55, *p* < 0.05, Figure 3E) and 48 h (P: −6.11 ± 1.18%, C: −12.44 ± 2.10%, Cohen’s d = 0.99, 95% CI: 1.61 to 11.05, *p* < 0.05, Figure 3E). No significant between-group differences were observed in the percentage change in SJ height at either 24 or 48 h post-exercise.

#### 3.4.4. Isokinetic Knee Strength Recovery

Isokinetic knee strength was measured pre-exercise and at 24 h and 48 h post-exercise. Relative peak torque and relative maximum work were assessed for both dominant and non-dominant limbs at 60°/s and 180°/s.

1. 60°/s angular velocity. As shown in Figure 4, at 60°/s the reduction in relative peak torque for the non-dominant limb was significantly smaller in the P group at 24 h (P: −10.08 ± 1.68%, C: −16.04 ± 2.29%, Cohen’s d = 0.79, 95% CI: 0.39 to 11.53, *p* < 0.05, Figure 4B) and 48 h (P: −5.15 ± 1.02%, C: −9.55 ± 1.45%, Cohen’s d = 0.94, 95% CI: 0.93 to 7.87, *p* < 0.05, Figure 4B) post-exercise. Similarly, the reduction in relative maximum work for the non-dominant limb was significantly smaller in the P group at 24 h (P: −11.30 ± 1.78%, C: −19.32 ± 3.13%, Cohen’s d = 0.84, 95% CI: 0.96 to 15.08, *p* < 0.05, Figure 4D) and 48 h (P: −6.73 ± 1.48%, C: −12.06 ± 1.95%, Cohen’s d = 0.82, 95% CI: 0.53 to 10.13, *p* < 0.05, Figure 4D). No significant between-group differences were observed for the dominant limb.
2. 180°/s angular velocity. As shown in Figure 4, at 180°/s the P group exhibited significantly smaller reductions in relative peak torque for the non-dominant limb at 24 h (P: −10.82 ± 1.45%, C: −15.85 ± 1.90%, Cohen’s d = 0.80, 95% CI: 0.35 to 9.71, *p* < 0.05, Figure 4F) and 48 h (P: −7.43 ± 1.56%, C: −12.68 ± 1.84%, Cohen’s d = 0.82, 95% CI: 0.52 to 9.98, *p* < 0.05, Figure 4F) post-exercise. The reduction in relative maximum work for the non-dominant limb was also significantly attenuated in the P group at 24 h (absolute values: P, 1.39 ± 0.05 J/kg; C, 1.15 ± 0.05 J/kg; P: −15.67 ± 3.27%, C: −29.61 ± 2.78%, Cohen’s d = 1.23, 95% CI: 5.53 to 22.35, p < 0.05, Figure 4H) and at 48 h (P: −9.49 ± 3.35%, C: −21.45 ± 3.29%, Cohen’s d = 0.96, 95% CI: 2.76 to 21.16, *p* < 0.05, Figure 4H). No significant between-group differences were found for the dominant limb.

### 3.5. Effects on Gut Microbiota

#### 3.5.1. Alpha Diversity

Alpha diversity of the gut microbiota, assessed using the Chao1 and Simpson indices, was analyzed from fecal samples collected pre- and post-intervention (BP: P group baseline, BC: C group baseline, AP: P group post-intervention, AC: C group post-intervention). Repeated-measures ANOVA revealed a significant group × time interaction for Chao1 (F(1, 26) = 5.96, *p* < 0.05, ηp^2^ = 0.19). Within-group comparisons showed that Chao1 decreased significantly in the P group (BP: 262.52 ± 16.01, AP: 211.76 ± 13.28, *p* < 0.05), whereas no significant change was observed in the C group (BC: 229.49 ± 20.00, AC: 222.11 ± 21.63, *p* > 0.05, Figure 5A). In contrast, the group × time interaction was not significant for the Simpson index (F(1,26) = 0.69, *p* > 0.05, ηp^2^ = 0.03, Figure 5B). Within-group comparisons showed that the Simpson index decreased in both the P group (BP: 0.95 ± 0.01, AP: 0.93 ± 0.01, *p* < 0.05) and the C group (BC: 0.95 ± 0.01, AC: 0.93 ± 0.01, *p* < 0.05, Figure 5B).

#### 3.5.2. Taxonomic Composition

Taxonomic composition at the phylum and family levels is presented in Figure 5C–F. Repeated-measures ANOVA showed no significant group × time interaction for Firmicutes (F(1,26) = 1.17, *p* > 0.05, ηp^2^ = 0.04), Lachnospiraceae (F(1,26) = 2.56, *p* > 0.05, ηp^2^ = 0.09), or Bifidobacteriaceae (F(1,26) = 0.47, *p* > 0.05, ηp^2^ = 0.02). Exploratory within-group comparisons showed that the relative abundance of Firmicutes (phylum) increased from 0.58 ± 0.03 to 0.65 ± 0.05, that of Lachnospiraceae (family) increased from 0.21 ± 0.03 to 0.32 ± 0.05, and that of Bifidobacteriaceae (family) increased from 0.02 ± 0.01 to 0.04 ± 0.01 in the P group (all *p* < 0.05, Figure 5D–F). No significant within-group changes in these taxa were observed in the C group. 

### 3.6. Sensitivity Analysis

To evaluate the robustness of findings under multiplicity adjustment, post hoc Holm adjustment was applied separately to the performance and recovery comparisons presented in Figure 2, Figure 3 and Figure 4, the two alpha-diversity interaction tests, and the three taxonomic interaction tests. Among the performance and recovery outcomes, two comparisons survived the Holm-adjusted significance threshold: 180°/s non-dominant limb maximum work at 24 h (Holm-adjusted α = 0.0023) and VAS at 24 h (Holm-adjusted α = 0.0024). For alpha diversity, the group × time interaction for Chao1 remained significant after Holm adjustment (adjusted *p* = 0.044), whereas the Simpson interaction was nonsignificant (adjusted *p* = 0.413). The interactions for Firmicutes, Lachnospiraceae, and Bifidobacteriaceae remained nonsignificant (adjusted *p* = 0.579, 0.364, and 0.579, respectively).

## 4. Discussion

This exploratory trial identified between-group differences in several performance and recovery outcomes. However, only muscle soreness at 24 h and non-dominant knee extension maximum work at 180°/s at 24 h remained significant after Holm adjustment within the performance and recovery comparison family. The remaining findings should therefore be considered hypothesis-generating. In the separately adjusted microbiome analyses, the Chao1 group × time interaction remained significant within the alpha-diversity comparison family, whereas the Simpson interaction and all three taxonomic interactions were nonsignificant after adjustment within their respective comparison families. Overall, these findings provide limited evidence for differences in selected recovery outcomes but do not establish broad benefits of probiotic supplementation or a microbiota-mediated mechanism.

The decline in athletic performance and lower-limb strength during detraining is well documented and is often attributed to diminished neuromuscular activation and fast-twitch fiber atrophy; similar observations have been made in longitudinal studies monitoring athletes during periods of reduced training supervision [6,7]. In the present study, no significant differences were observed between the P and C groups during detraining in Wingate test parameters (relative peak power, mean power and fatigue index), IMTP peak force ratio, or isokinetic knee strength measures (including relative peak work and torque of both dominant and non-dominant limbs at 60°/s and 180°/s). These metrics primarily reflect the instantaneous power or maximal strength of isolated lower-limb muscle groups, which are highly dependent on neural excitability, high-threshold motor-unit recruitment and maximal output of energy systems. These observations align with several previous studies. Przewłócka et al. [18] reported that probiotics combined with vitamin D3 had no effect on peak power in anaerobic tests. Mazur-Kurach et al. [23] observed that while probiotics improved recovery and aerobic performance, their effect on short-duration power metrics such as maximal anaerobic output was limited—potentially due to enhanced post-exercise lactate clearance rather than direct improvements in power. Similarly, Salleh et al. [24] observed no strength improvement in badminton athletes supplemented with Lactobacillus casei Shirota. Thus, the present study did not provide evidence that probiotic supplementation differentially affected the reported maximal-strength or Wingate outcomes across the intervention period.

In contrast, the P group showed significantly better performance retention in tests of integrated power (jumps) and sport-specific agility (shuttle test) (*p* < 0.05). However, these between-group differences did not remain significant after Holm adjustment and should therefore be considered hypothesis-generating. Le Pellec and Maton [25] demonstrated that jumping performance depends on integrated central nervous system (CNS) anticipatory control and multi-joint coordination. Other studies have shown that neuromuscular control efficiency and muscle–tendon interaction differentiate superior jumping ability [26,27], and these findings are further supported by Lazaridis et al. [28]. In contrast, isokinetic knee strength primarily reflects muscle contractile properties, as confirmed by Wrucke et al. [29] using transcranial magnetic stimulation. Although these differences between performance tests may provide context for the observed pattern, the present findings do not establish that probiotic supplementation has preferential effects on complex performance tasks rather than maximal-strength measures. The specific neuromuscular processes underlying these observations were not directly assessed. Previous studies have suggested that gut microbiota may influence central nervous system and muscle function potentially through gut–brain–muscle communication [11]. However, this interpretation remains speculative because the relevant physiological and molecular pathways were not directly assessed in the present study. Previous studies have also suggested that probiotics may modulate immune and inflammatory responses [15]. Such changes could potentially contribute to the maintenance of neuromuscular function during recovery. However, inflammatory markers and neuromuscular junction function were not directly measured in the present study, and therefore this proposed pathway cannot be confirmed from our data. Furthermore, improved gut health from probiotic supplementation may enhance nutrient absorption and optimize energy metabolism. Huang et al. [15] reported that L. plantarum supplementation improved energy metabolism and concurrently enhanced jumping performance. Probiotic-related changes in microbial metabolites, including SCFAs, have also been proposed to influence metabolic pathways such as AMPK–PGC-1α signaling [30] and glucose metabolism [31]. These pathways could potentially contribute to exercise recovery by affecting mitochondrial function and energy metabolism. However, inflammatory markers, SCFA concentrations, nutrient absorption, and neural or metabolic signaling pathways were not measured in the present study. These mechanisms therefore remain hypothetical and cannot explain the observed performance findings.

To model the physiological stress associated with an early return to training, a standardized muscle-damaging exercise protocol consisting of 200 weighted vertical jumps was used to induce DOMS and EIMD [22]. Before adjustment for multiple comparisons, the reductions in SJ relative peak force and RFD at 24 h and 48 h post-exercise were smaller in the P group than in the C group. A smaller reduction in the IMTP peak force ratio was also observed in the P group at 24 h. However, none of these comparisons remained significant after Holm adjustment; therefore, these findings should be considered hypothesis-generating. Similarly, 6-week supplementation with Lactobacillus paracasei PS23 has been reported to attenuate post-EIMD declines in RFD and force in physically active individuals [17]. Notably, the lack of a between-group difference in SJ height in the present study may be attributable to its multifactorial nature, including movement technique, lower-limb coordination and elastic rebound [32].

Isokinetic testing showed an exploratory pattern involving the non-dominant limb. Before adjustment for multiple comparisons, the P group exhibited significantly smaller reductions in relative peak torque and relative maximum work in the non-dominant limb at both 60°/s and 180°/s at 24 h and 48 h post-exercise, whereas no significant between-group differences were observed in the dominant limb. After Holm adjustment, only non-dominant knee extension maximum work at 180°/s at 24 h remained significant. This result provides limited evidence of a difference in this specific recovery outcome but does not establish a general benefit for isokinetic knee-strength recovery. Similar limb-specific findings have been reported in other intervention studies [33,34], although the underlying reasons for such differences between limbs remain unclear. Further adequately powered studies with prespecified limb-related comparisons are needed to determine whether this finding is reproducible. The observed improvements in recovery-related outcomes may have several possible explanations [30,35], but the present study did not directly assess the underlying inflammatory, oxidative, or metabolic pathways. Therefore, these potential mechanisms should be considered speculative and interpreted cautiously.

Muscle soreness and exercise-induced muscle damage have been associated with elevated inflammatory and oxidative stress biomarkers [36,37]. In the present study, the P group exhibited markedly lower DOMS scores at 24 h post-exercise (Cohen’s d = −1.17), and this difference remained significant after Holm adjustment. The P group also showed smaller CK elevation at both 24 h and 48 h (Cohen’s d = −0.89 and −0.87, respectively). However, these CK comparisons did not remain significant after Holm adjustment and should therefore be considered hypothesis-generating. Thus, the findings do not establish a consistent supplementation effect across subjective muscle soreness and biochemical markers of muscle damage. Jäger et al. [38] also found that Bacillus coagulans GBI-30 supplementation reduced muscle soreness and accelerated recovery; multi-strain probiotic supplementation has been shown to alleviate muscle soreness and leg heaviness in elite rugby players [39], and Lee et al. [17] reported that Lactobacillus paracasei PS23 lowered soreness and serum CK after exercise-induced muscle damage. Previous studies have also suggested that selected probiotic strains may influence muscle recovery, inflammation, and oxidative stress [17,35]. However, the effects of probiotics may depend on the strain, population, supplementation duration, and exercise protocol. Inflammatory cytokines and oxidative stress markers were not measured in the present study; therefore, the mechanisms underlying the observed muscle-soreness result cannot be determined from our data.

Gut microbiota analysis showed a greater reduction in Chao1 richness in the probiotic group than in the placebo group, with the group × time interaction remaining significant after Holm adjustment. In contrast, no significant interaction was observed for the Simpson index. These findings indicate a differential change in estimated microbial richness rather than a consistent change across alpha-diversity measures. The biological significance of the reduction in Chao1 richness remains uncertain and should not, by itself, be interpreted as a beneficial or adverse microbial adaptation. At a descriptive level, the within-group increases in the relative abundances of Firmicutes, Lachnospiraceae, and Bifidobacteriaceae are partly compatible with previous reports of changes in gut microbial composition following probiotic intake [40]. However, none of the three taxa showed a significant group × time interaction, either before or after multiplicity adjustment; therefore, these observations do not establish an intervention effect. Firmicutes and Lachnospiraceae include members capable of producing SCFAs [41,42], which have been proposed to influence skeletal muscle metabolism [43,44]. Previous research has also suggested that microbiota-derived metabolites may contribute to muscle-related effects of probiotics [17]. These possibilities remain hypothetical in the present study, as relative-abundance changes cannot establish increased SCFA production, and neither SCFA concentrations nor microbiota–performance associations were assessed. Consequently, the microbiome findings do not establish a mechanism underlying the observed performance or recovery outcomes.

Several limitations should be acknowledged. First, this trial is exploratory in nature, with most findings regarded as hypothesis-generating rather than established treatment effects, as only two of the numerous between-group comparisons survived Holm multiplicity adjustment. Second, the relatively small sample size and inclusion of national-level university volleyball athletes may limit statistical power, precision, and generalizability. The absence of a formal a priori sample-size calculation and the presence of multiple comparisons further warrant cautious interpretation, with effect sizes and 95% confidence intervals considered alongside P values. Third, microbiome analysis was limited to the phylum and family levels, without genus/species-level characterization, functional prediction, or microbiota–performance correlation analyses. SCFAs, inflammatory cytokines, and oxidative stress markers were also not measured; therefore, the microbiota findings and proposed mechanisms should be regarded as associative and hypothesis-generating rather than causal. Fourth, the standardized simulated return-to-training stressor did not fully replicate the physiological demands of an actual return-to-training programme, and the single-blind design may have introduced some risk of bias during supplement allocation. Finally, dietary intake was not quantitatively monitored, and retrospective registration without prospectively prespecified primary and secondary outcomes or a detailed statistical analysis plan means that selective reporting cannot be completely excluded. Future studies should use larger, prospectively registered cohorts with more comprehensive microbiome and physiological assessments.

The present findings provide a rationale for further investigation of probiotic supplementation during training transitions, but are insufficient to support a specific supplementation recommendation for performance retention or recovery.

## 5. Conclusions

This exploratory trial identified lower muscle soreness and a smaller reduction in non-dominant knee extension maximum work at 180°/s at 24 h in the probiotic group than in the placebo group; both findings remained significant after Holm adjustment. Other performance and recovery findings should be considered hypothesis-generating. The microbiome results do not establish an underlying mechanism. Confirmation in larger, prospectively registered trials with prespecified outcomes is required.

## Figures and Tables

**Figure 1:**
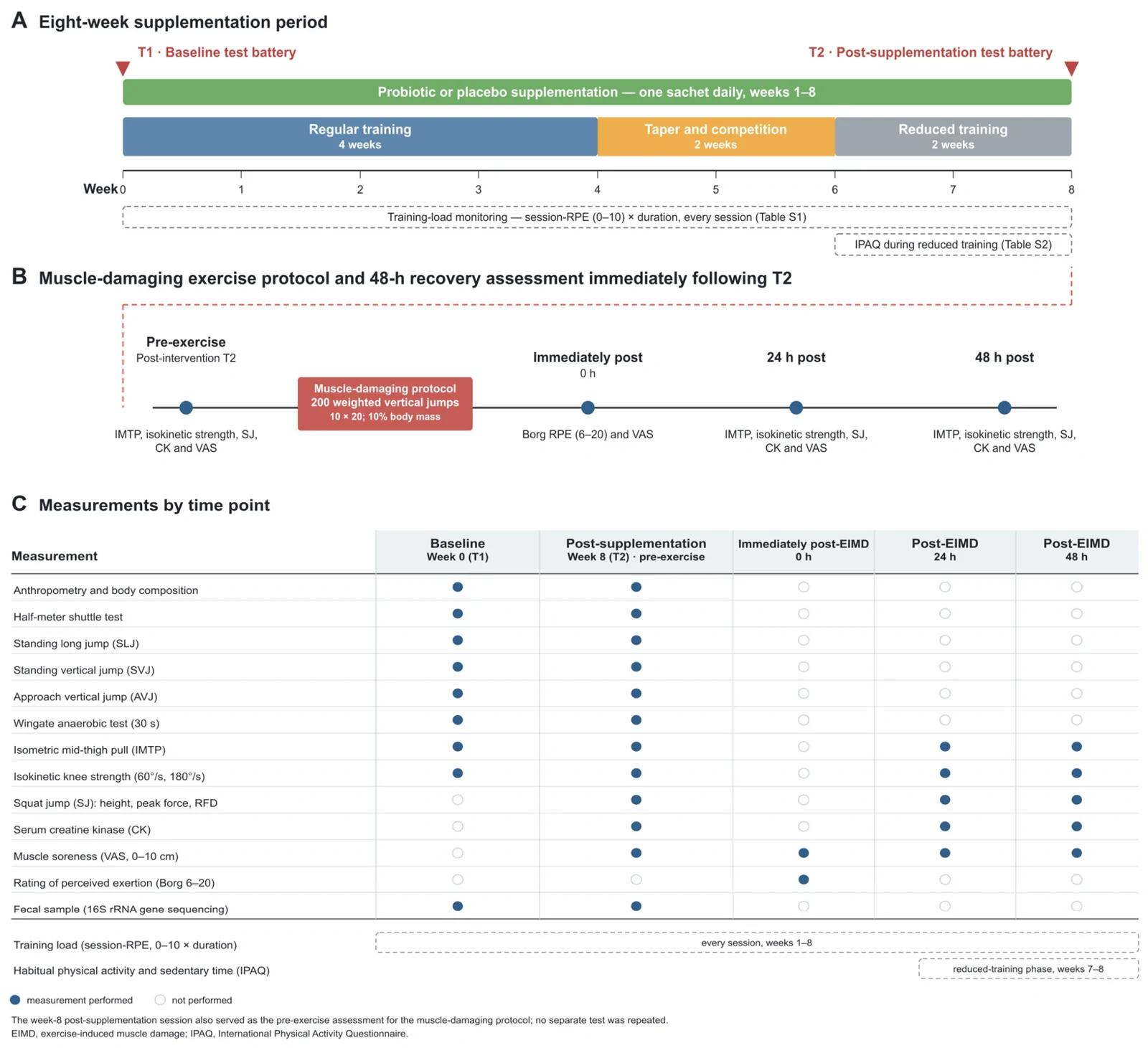
Study design and measurement schedule. (**A**) The 8-week supplementation period comprised 4 weeks of regular training, 2 weeks of tapering and competition, and 2 weeks of reduced training. Participants consumed one sachet of probiotic or placebo daily throughout the intervention. Training load was monitored during every training session, and habitual physical activity and sedentary time were assessed during the reduced-training period. (**B**) Immediately after the week-8 post-supplementation assessment, participants completed a muscle-damaging exercise protocol consisting of 200 weighted vertical jumps. IMTP, isokinetic knee strength, SJ, serum CK, and muscle soreness were assessed before the exercise protocol and at 24 and 48 h post-exercise. RPE and muscle soreness were assessed immediately after the protocol. (**C**) Measurements performed at each study time point. Anthropometry and body composition were assessed at both baseline and post-supplementation. SJ was assessed only at pre-exercise and at 24 and 48 h post-exercise. CK, creatine kinase; EIMD, exercise-induced muscle damage; IMTP, isometric mid-thigh pull; IPAQ, International Physical Activity Questionnaire; RFD, rate of force development; RPE, rating of perceived exertion; SJ, squat jump; VAS, visual analog scale.

**Figure 2:**
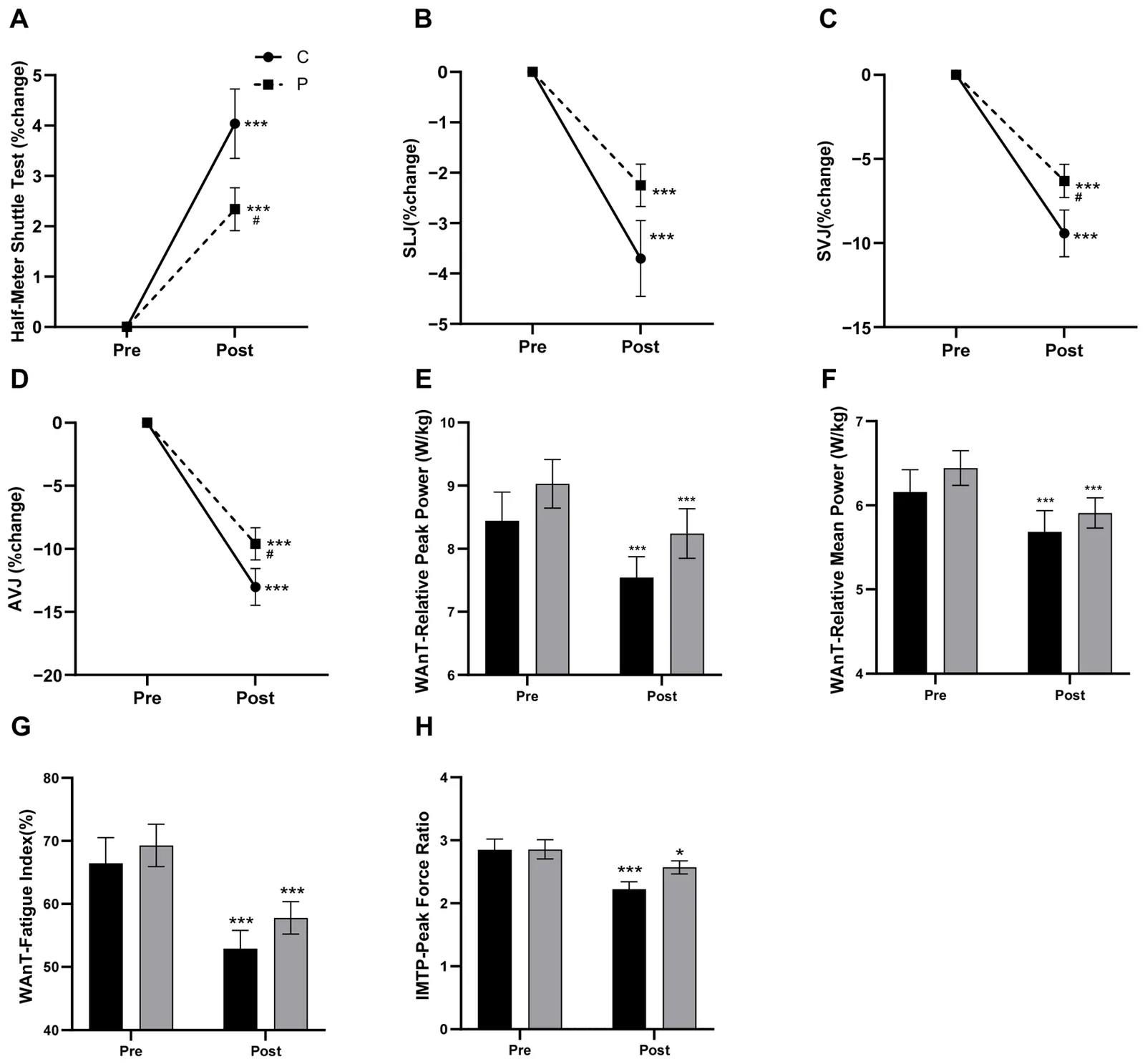
Changes in sport-specific performance, anaerobic power and maximal strength before and after the 8-week intervention in the placebo (C) and probiotic (P) groups. (**A**) Half-meter shuttle test time; (**B**) standing long jump distance; (**C**) standing vertical jump (SVJ) height; (**D**) approach vertical jump (AVJ) height; (**E**) Wingate test (WAnT) relative peak power; (**F**) WAnT relative mean power; (**G**) WAnT fatigue index; (**H**) IMTP peak force ratio. Data are expressed as mean ± SEM. For outcomes expressed as raw values (**E**–**H**), repeated-measures ANOVA was performed to assess group × time effects; for outcomes expressed as percentage changes from baseline (**A**–**D**), between-group comparisons were conducted using independent-samples *t*-tests. # indicates a significant between-group difference (# *p* < 0.05). * indicates significant within-group differences compared with pre-intervention (paired t-tests with Bonferroni correction: * *p* < 0.05, *** *p* < 0.001).

**Figure 3:**
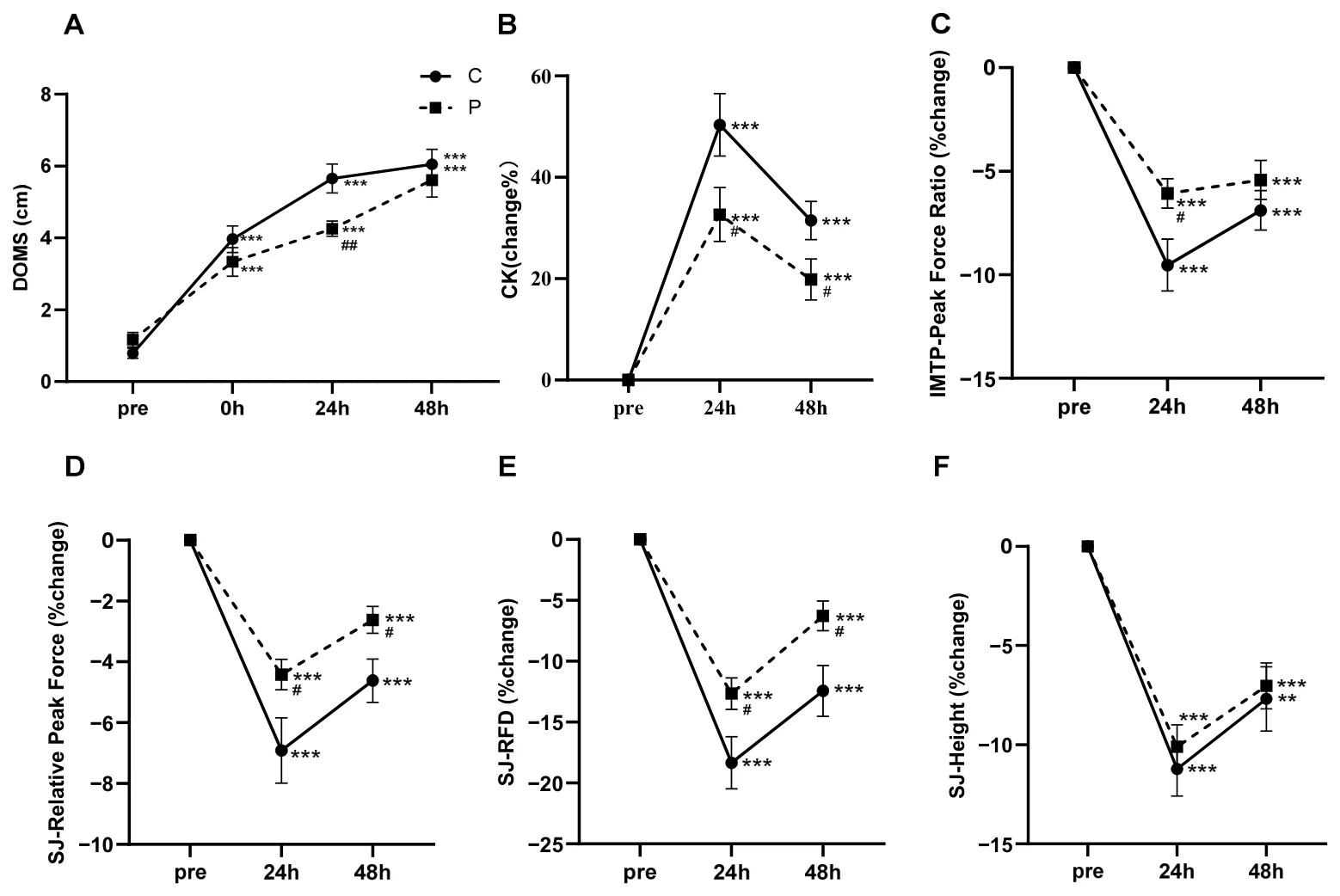
Muscle soreness, creatine kinase (CK) levels and recovery of lower-limb strength and power after exercise-induced muscle damage. (**A**) Muscle soreness assessed by visual analog scale (VAS); (**B**) percentage change in serum CK; (**C**) percentage change in the isometric mid-thigh pull (IMTP) peak force ratio; (**D**) percentage change in squat jump (SJ) relative peak force; (**E**) percentage change in SJ rate of force development (RFD); (**F**) percentage change in SJ height. Data are expressed as mean ± SEM. Muscle soreness (VAS, A) was analyzed using repeated-measures ANOVA to assess group × time effects. All other indicators (**B**–**F**) were compared between groups using independent-samples *t*-tests. # indicates a significant between-group difference (# *p* < 0.05, ## *p* < 0.01). Asterisks indicate significant within-group differences compared with pre-exercise (** *p* < 0.01, *** *p* < 0.001).

**Figure 4:**
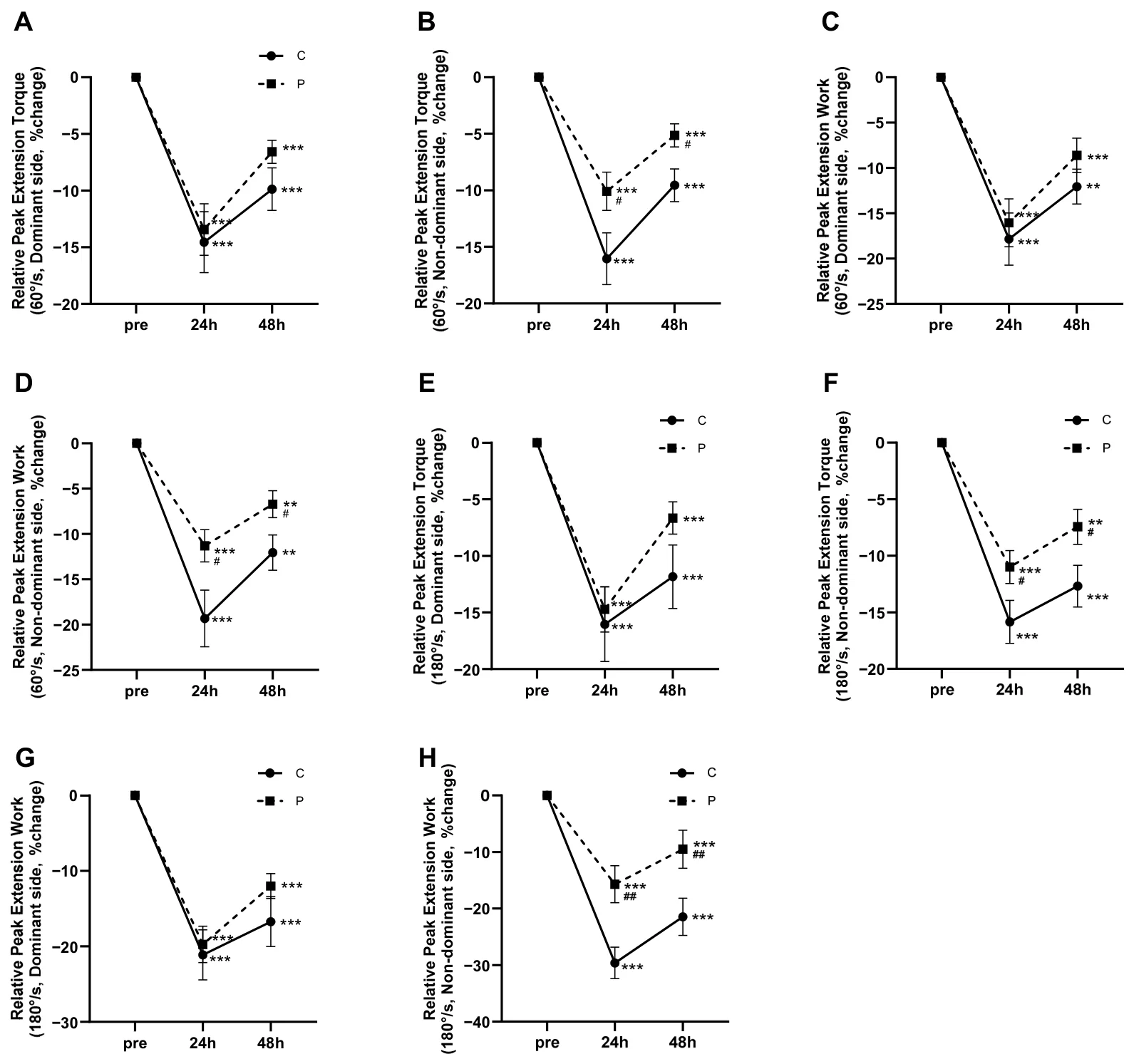
Recovery of isokinetic knee strength following exercise-induced muscle damage in the placebo (**C**) and probiotic (**P**) groups. (**A**,**B**) Relative peak torque at 60°/s in the dominant (**A**) and non-dominant (**B**) limbs; (**C**,**D**) relative maximum work at 60°/s in the dominant (**C**) and non-dominant (**D**) limbs; (**E**,**F**) relative peak torque at 180°/s in the dominant (**E**) and non-dominant (**F**) limbs; (**G**,**H**) relative maximum work at 180°/s in the dominant (**G**) and non-dominant (**H**) limbs. Data are expressed as mean ± SEM. Between-group comparisons of percentage changes from pre-exercise were performed using independent-samples t-tests. # indicates a significant between-group difference (# *p* < 0.05, ## *p* < 0.01). Asterisks indicate significant within-group differences compared with pre-exercise (** *p* < 0.01, *** *p* < 0.001).

**Figure 5:**
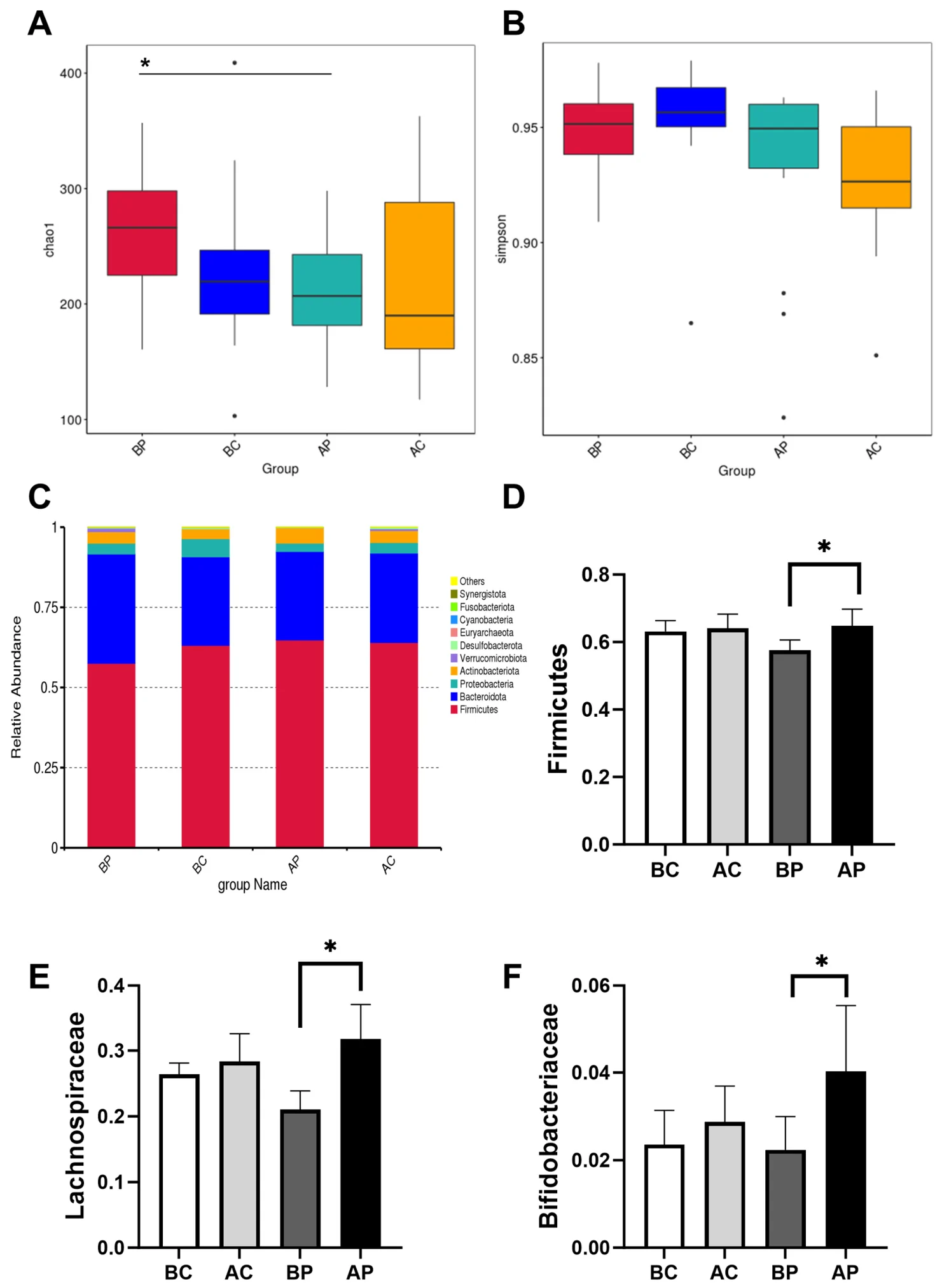
Effects of probiotic supplementation on gut microbiota. (**A**) Chao1 index of alpha diversity; (**B**) Simpson index of alpha diversity; (**C**) stacked bar chart of taxonomic composition; (**D**) relative abundance of Firmicutes at the phylum level; (**E**) relative abundance of Lachnospiraceae at the family level; (**F**) relative abundance of Bifidobacteriaceae at the family level. BP: P group baseline; BC: C group baseline; AP: P group post-intervention; AC: C group post-intervention. Data are expressed as mean ± SEM. Group × time interactions were examined using repeated-measures ANOVA. Exploratory within-group pre–post comparisons were performed using paired-samples *t*-tests, * *p* < 0.05.

**Table 1:** Anthropometric characteristics of participants in the placebo (C) and probiotic (P) groups before the 8-week intervention.

Variable	C	P
Body mass (kg)	73 ± 11	73 ± 11
Skeletal muscle mass (kg)	32.50 ± 1.99	32.85 ± 2.15
Body fat mass (kg)	14.94 ± 1.49	13.44 ± 1.61
Soft lean mass (kg)	54.65 ± 3.13	55.62 ± 3.32
Fat-free mass (kg)	58.02 ± 3.29	59.24 ± 3.49
BMI (kg/m^2^)	21.56 ± 0.40	21.81 ± 0.63
Body fat percentage (%)	19.41 ± 1.57	19.25 ± 2.07
WHR	0.850 ± 0.005	0.850 ± 0.008

Body mass is presented as mean ± SD; all other variables are presented as mean ± SEM. C, placebo group (n = 14); P, probiotic group (n = 14). Between-group comparisons were performed using independent-samples *t*-tests. No significant differences were detected between groups (*p* > 0.05). BMI, body mass index; WHR, waist-to-hip ratio; SD, standard deviation; SEM, standard error of the mean.

**Table 2:** Isokinetic knee strength (relative peak torque and relative maximum work) at 60°/s and 180°/s in the dominant and non-dominant limbs, pre- and post-intervention.

Velocity	Limb	Time	Relative Max Work (J/kg)		Relative Peak Torque (Nm/kg)	
			C	P	C	P
60°/s	Dominant	Pre	2.14 ± 0.08	2.27 ± 0.07	2.74 ± 0.11	2.84 ± 0.10
60°/s	Dominant	Post	1.93 ± 0.07 ***	1.99 ± 0.07 ***	2.42 ± 0.10 ***	2.56 ± 0.10 ***
60°/s	Non-dominant	Pre	2.02 ± 0.07	2.15 ± 0.07	2.68 ± 0.11	2.70 ± 0.10
60°/s	Non-dominant	Post	1.83 ± 0.07 ***	1.90 ± 0.06 ***	2.43 ± 0.11 ***	2.42 ± 0.07 ***
180°/s	Dominant	Pre	1.85 ± 0.07	1.87 ± 0.06	2.22 ± 0.11	2.18 ± 0.08
180°/s	Dominant	Post	1.63 ± 0.05 ***	1.72 ± 0.06 ***	1.99 ± 0.08 ***	2.07 ± 0.07 **
180°/s	Non-dominant	Pre	1.77 ± 0.07	1.81 ± 0.04	2.11 ± 0.09	2.19 ± 0.07
180°/s	Non-dominant	Post	1.58 ± 0.05 ***	1.65 ± 0.05 ***	1.89 ± 0.08 ***	2.00 ± 0.07 ***

Data are mean ± SEM. C, placebo group (n = 14); P, probiotic group (n = 14). Within-group comparisons (post vs. pre-intervention) were performed using repeated-measures ANOVA followed by Bonferroni-adjusted pairwise comparisons. ** *p* < 0.01 and *** *p* < 0.001 vs. pre-intervention within the same group.

## Data Availability

The data presented in this study are available on request from the corresponding author due to privacy restrictions related to athlete information.

## Supplementary Materials

The following supporting information can be downloaded at: https://www.mdpi.com/article/10.3390/nu18193203/s1. Section S1. Training Load Monitoring (session-RPE). Section S2. Daily Physical Activity Assessment (IPAQ). Section S3. Summary of Load and Activity Equivalence Between Groups. Table S1: Mean weekly training load (session-RPE, arbitrary units) in the placebo (C) and probiotic (P) groups across distinct phases of the training cycle (mean ± SEM).; Table S2: Habitual physical activity and sedentary time in the placebo (C) and probiotic (P) groups during the reduced-training phase (mean ± SEM); Table S3: Non-percentage performance values before and after the 8-week intervention in the placebo and probiotic groups; Table S4: Non-percentage values of post-exercise recovery and muscle-damage outcomes following the weighted-jump protocol. Table S5: Body composition before and after the intervention; Figure S1: Schematic diagram of the half-meter shuttle test.

## Abbreviations

The following abbreviations are used in this manuscript:

AMPK	AMP-activated protein kinase
ANOVA	Analysis of variance
AVJ	Approach vertical jump
CFU	Colony-forming units
CK	Creatine kinase
CNS	Central nervous system
DOMS	Delayed-onset muscle soreness
EIMD	Exercise-induced muscle damage
ELISA	Enzyme-linked immunosorbent assay
GLP-1	Glucagon-like peptide-1
IMTP	Isometric mid-thigh pull
IPAQ	International Physical Activity Questionnaire
NF-κB	Nuclear factor kappa-B
PGC-1α	Peroxisome proliferator-activated receptor gamma coactivator 1-alpha
SCFAs	Short-chain fatty acids
SEM	Standard error of the mean
SJ	Squat jump
SLJ	Standing long jump
SVJ	Standing vertical jump
VAS	Visual analog scale
