# Supplementary material for: Effects of Probiotic Supplementation on Performance Retention During Detraining and Recovery After Simulated Return-to-Training Stress in College Volleyball Players: An Exploratory Randomized, Single-Blind, Placebo-Controlled Trial
Source: Nutrients. 2026 Sep 28;18(19):3203. doi: 10.3390/nu18193203 (PMC13667075; doi:10.3390/nu18193203)
Supplement: Supplementary file 1 [file nutrients-18-03203-s001.zip › nutrients-4474125-supplementary.pdf]

Supplementary Materials

Effects of Probiotic Supplementation on Performance Retention During Detraining and Recovery After Simulated Return-to-Training Stress in College Volleyball Players: An Exploratory Randomized, Single-Blind, Placebo-Controlled Trial

Jin Liu 1,3,†, Kai Zhao 2,3,†, Chenzhe Liu 1,3*, Zhichen Liu1,3 and Wei Jiang2

Contents

Section S1. Training Load Monitoring (session-RPE)

Section S2. Daily Physical Activity Assessment (IPAQ)

Section S3. Summary of Load and Activity Equivalence Between Groups

Table S1. Mean weekly training load across phases of the training cycle

Table S2. Habitual physical activity and sedentary time during detraining

Table S3: Non-percentage performance values before and after the 8-week intervention.

Table S4: Non-percentage values of post-exercise recovery and muscle-damage outcomes following the weighted-jump protocol.

Table S5. Body composition before and after the intervention.

Figure S1: Schematic diagram of the half-M-shaped shuttle run test

S1. Training Load Monitoring (Session-RPE)

S1.1. Calculation Method

Athlete training load was quantified using the session-rating of perceived exertion (session-RPE) method. The load for any single training session or competition was calculated as the product of its duration (min) and the athlete's self-reported rating of perceived exertion (RPE; 0–10 scale), collected within 30 min after the session:

$$\textit{Session load (AU)} = \textit{duration (min)} \times \textit{RPE (0–10)}$$

The total load for a specific phase (e.g., training block, taper, competition, detraining) was computed as the sum of all individual session loads within that period. For standardized comparison across phases of different durations, loads are reported as weekly equivalents. When a phase lasted fewer than 7 days, the daily average was scaled to a 7-day week:

$$\textit{Equivalent weekly load} = (\textit{Total phase load} \div \textit{number of days in phase}) \times 7$$

S1.2. Results

The 8-week intervention comprised three successive phases: a 4-week regular training phase, a 2-week combined taper and competition phase, and a 2-week detraining phase. The mean weekly training loads for the placebo (C) and probiotic (P) groups across distinct periods of the training cycle are summarized in Table S1. No significant between-group differences were observed in any phase (all $p > 0.05$).

Table S1. Mean weekly training load (session-RPE, arbitrary units) in the placebo (C) and probiotic (P) groups across distinct phases of the training cycle (mean ± SEM).

Phase	C (n = 14)	P (n = 14)	p-value
Training	8181.1 ± 225.48	7641.3 ± 289.69	> 0.05
Taper	4404.1 ± 143.43	4181.4 ± 108.80	> 0.05

Competition	6717.3 ± 181.49	6665.0 ± 182.54	> 0.05
Detraining	1615.9 ± 60.17	1639.4 ± 73.11	> 0.05

Values are mean ± SEM in arbitrary units (AU) of the session-RPE method. C, placebo group; P, probiotic group. Between-group comparisons were performed using independent-samples *t*-tests (or Mann–Whitney U tests where appropriate). AU, arbitrary units; RPE, rating of perceived exertion; SEM, standard error of the mean.

S2. Daily Physical Activity Assessment (IPAQ)

S2.1. Calculation Method

Habitual physical activity during periods without prescribed training was assessed using the long-form International Physical Activity Questionnaire (IPAQ). Self-reported time spent in activities of light (LPA), moderate (MPA) and vigorous (VPA) intensity was converted to metabolic equivalent of task minutes per week (MET-min·week^{-1}) following standard IPAQ scoring. Composite indices were derived as follows:

$$\textit{Moderate-to-Vigorous Physical Activity (MVPA)} = MPA + VPA$$

$$\textit{Total Physical Activity (TPA)} = LPA + MVPA$$

Sedentary time (ST) was recorded directly as minutes per week (min·week^{-1}).

S2.2. Results

Daily physical activity levels and sedentary time for both groups during the detraining period are presented in Table S2. No significant between-group differences were observed (all $p > 0.05$), confirming that habitual activity during unstructured periods was matched between groups.

Table S2. Habitual physical activity and sedentary time in the placebo (C) and probiotic (P) groups during the reduced-training phase (mean ± SEM).

Variable	C (n = 14)	P (n = 14)	p-value
Light PA (MET-min·week^{-1})	379.3 ± 10.23	341.4 ± 8.15	> 0.05
Moderate PA (MET-min·week^{-1})	72.9 ± 1.51	62.1 ± 1.86	> 0.05
Vigorous PA (MET-min·week^{-1})	19.8 ± 0.43	18.3 ± 0.29	> 0.05
Moderate-to-Vigorous PA (MET-min·week^{-1})	92.7 ± 1.61	80.4 ± 1.89	> 0.05
Total PA (MET-min·week^{-1})	472.0 ± 10.02	421.7 ± 8.03	> 0.05
Sedentary time (min·week^{-1})	632.5 ± 19.18	648.7 ± 19.76	> 0.05

Values are mean ± SEM. C, placebo group; P, probiotic group. Light PA, moderate PA, vigorous PA, MVPA and total PA are expressed in metabolic equivalent of task minutes per week (MET-min·week^{-1}). Sedentary time is expressed as minutes per week (min·week^{-1}). Between-group comparisons were performed using independent-samples *t*-tests (or Mann–Whitney U tests where appropriate). PA, physical activity; MVPA, moderate-to-vigorous physical activity; MET, metabolic equivalent of task; SEM, standard error of the mean.

S3. Summary of Load and Activity Equivalence Between Groups

The data presented in Sections S1 and S2 indicate that the placebo (C) and probiotic (P) groups were exposed to comparable external training loads across all phases of the intervention (training, taper, competition and detraining) and reported similar levels of habitual physical activity during the detraining period. These findings reduce the likelihood that differences in training load or habitual physical activity explain the observed between-group patterns. However, they do not by themselves establish that the observed differences were caused by probiotic supplementation.

Table S3. Non-percentage performance values before and after the 8-week intervention in the placebo and probiotic groups.

Outcome	Group	Pre-intervention	Post-intervention
Half-meter shuttle test time (s)	C	16.57 ± 0.24	17.23 ± 0.24
	P	16.71 ± 0.30	17.10 ± 0.31
SLJ distance (cm)	C	228.14 ± 8.13	219.70 ± 1.71
	P	221.35 ± 6.90	216.37 ± 0.93
SVJ height (cm)	C	49.88 ± 3.20	45.18 ± 0.69
	P	48.27 ± 2.71	45.23 ± 0.48
AVJ height (cm)	C	58.21 ± 2.98	50.86 ± 3.13
	P	61.14 ± 3.63	54.99 ± 3.00

Data are presented as mean ± SEM. C, placebo group; P, probiotic group; SLJ, standing long jump; SVJ, standing vertical jump; AVJ, approach vertical jump.

Table S4. Non-percentage values of post-exercise recovery and muscle-damage outcomes following the weighted-jump protocol.

Outcome	Group	pre-exercise	24 h	48 h
**CK (ng/mL)**	C	1.48 ± 0.30	2.23 ± 0.09	1.95 ± 0.06
	P	1.14 ± 0.21	1.52 ± 0.06	1.37 ± 0.05
**IMTP peak force ratio**	C	2.89 ± 0.15	2.64 ± 0.15	2.71 ± 0.15
	P	2.99 ± 0.16	2.78 ± 0.15	2.84 ± 0.18
**SJ relative peak force (N/kg)**	C	2.46 ± 0.06	2.29 ± 0.03	2.35 ± 0.02
	P	2.40 ± 0.06	2.30 ± 0.03	2.34 ± 0.01
**SJ RFD (N/s)**	C	10806.91 ± 2469.44	8824.92 ± 230.19	9462.53 ± 226.95
	P	8366.55 ± 864.77	7307.34 ± 107.09	7855.35 ± 98.73
**SJ height (m)**	C	0.33 ± 0.02	0.29 ± 0.02	0.30 ± 0.02
	P	0.34 ± 0.03	0.30 ± 0.02	0.31 ± 0.02
**Knee extension relative peak torque at 60° /s, dominant limb (N • m/kg)**	C	2.69 ± 0.18	2.38 ± 0.24	2.48 ± 0.20
	P	2.53 ± 0.18	2.28 ± 0.18	2.40 ± 0.17
**Knee extension relative peak torque at 60° /s, non-dominant limb (N • m/kg)**	C	2.47 ± 0.17	2.08 ± 0.06	2.24 ± 0.04
	P	2.44 ± 0.07	2.19 ± 0.04	2.31 ± 0.02
**Knee extension relative peak torque at 180° /s, dominant limb (N • m/kg)**	C	2.20 ± 0.17	1.95 ± 0.13	2.06 ± 0.14
	P	2.18 ± 0.15	1.86 ± 0.15	2.07 ± 0.17

**Knee extension relative peak torque at 180°/s, non-dominant limb (N • m/kg)**	C	2.12 ± 0.09	1.79 ± 0.04	1.85 ± 0.04
	P	2.11 ± 0.15	1.88 ± 0.03	1.95 ± 0.03
**Knee extension relative maximum work at 60°/s, dominant limb (J/kg)**	C	2.12 ± 0.10	1.80 ± 0.14	1.91 ± 0.12
	P	1.94 ± 0.09	1.71 ± 0.11	1.83 ± 0.09
**Knee extension relative maximum work at 60°/s, non-dominant limb (J/kg)**	C	1.78 ± 0.07	1.43 ± 0.06	1.56 ± 0.03
	P	1.99 ± 0.08	1.76 ± 0.04	1.85 ± 0.03
**Knee extension relative maximum work at 180°/s, dominant limb (J/kg)**	C	1.75 ± 0.08	1.41 ± 0.09	1.49 ± 0.08
	P	1.80 ± 0.08	1.50 ± 0.08	1.58 ± 0.09
**Knee extension relative maximum work at 180°/s, non-dominant limb (J/kg)**	C	1.64 ± 0.07	1.15 ± 0.05	1.29 ± 0.05
	P	1.64 ± 0.06	1.39 ± 0.05	1.49 ± 0.06

Data are presented as mean ± SEM. C, placebo group; P, probiotic group; CK, creatine kinase; IMTP, isometric mid-thigh pull; SJ, squat jump; RFD, rate of force development. The IMTP peak force ratio was calculated as (absolute peak force − body weight force) divided by body weight force and is dimensionless.

Table S5. Body composition before and after the intervention.

Variable	Before		After	
	C	P	C	P
Body mass (kg)	73 ± 11	73 ± 11	74 ± 10	74 ± 10
Skeletal muscle mass (kg)	32.50 ± 1.99	32.85 ± 2.15	32.42 ± 2.02	33.40 ± 2.07
Body fat mass (kg)	14.94 ± 1.49	13.44 ± 1.61	15.56 ± 1.24	13.74 ± 1.51
Soft lean mass (kg)	54.65 ± 3.13	55.62 ± 3.32	54.74 ± 3.04	56.49 ± 3.14
Fat-free mass (kg)	58.02 ± 3.29	59.24 ± 3.49	58.17 ± 3.23	60.16 ± 3.31
BMI (kg/m²)	21.56 ± 0.40	21.81 ± 0.63	21.77 ± 0.42	22.05 ± 0.53
Body fat percentage (%)	19.41 ± 1.57	19.25 ± 2.07	21.54 ± 1.78	18.92 ± 1.97
WHR	0.850 ± 0.005	0.850 ± 0.008	0.857 ± 0.005	0.853 ± 0.008

Body mass is presented as mean ± SD; all other variables are presented as mean ± SEM. C, placebo group (n = 14); P, probiotic group (n = 14); BMI, body mass index; WHR, waist-to-hip ratio; SD, standard deviation; SEM, standard error of the mean.

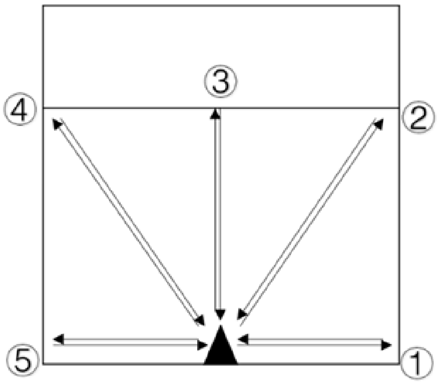

Figure S1. Schematic diagram of the half-meter shuttle test. Participants started at the baseline midpoint and completed the course in the order shown (1 → start → 2 → start → 3 → start → 4 → start → 5 → start), touching each marker plate with their foot.
